# Microencapsulated *Akkermansia muciniphila* alleviates acute lung injury in juvenile mice by protecting intestinal barrier

**DOI:** 10.3389/fcimb.2026.1723364

**Published:** 2026-03-03

**Authors:** Yumeng Chen, Zhenghai He, Xiaowen Shi, Jun Zhang, Lejiao Mao, Zhaoxia Lu, Zhen Zou, Chengzhi Chen, Xia Qin, Yishi Li

**Affiliations:** 1 Department of Pulmonary and Critical Care Medicine, The First Affiliated Hospital of Chongqing Medical University, Chongqing, China; 2 Key Laboratory of Clinical Laboratory Diagnostics, Ministry of Education, College of Laboratory Medicine, Chongqing Medical University, Chongqing, China; 3 Department of Pharmacy, The First Affiliated Hospital of Chongqing Medical University, Chongqing, China; 4 Department of Occupational and Environmental Health, School of Public Health, Chongqing Medical University, Chongqing, China

**Keywords:** acute lung injury, *Akkermansia muciniphila*, gut-lung axis, juvenile mice, microcapsule

## Abstract

**Introduction:**

The gut-lung axis plays a critical role in the pathogenesis of acute lung injury (ALI). While intestinal microbiota, particularly *Akkermansia muciniphila* (AKK), has been linked to the regulation of ALI in adult murine model, its impact on juvenile hosts, who exhibit heightened susceptibility to lipopolysaccharide (LPS)-induced ALI, remains poorly understood. Moreover, despite microencapsulation enhancing the probiotic gastrointestinal survival and colonization of probiotics, the therapeutic potential of microencapsulated AKK (AKK-MC) in juvenile murine ALI has not been explored.

**Methods:**

In this study, juvenile mice were orally gavaged with live AKK or AKK-MC for 14 days, with LPS-induced ALI established on day 11. Lung tissues were analyzed for morphological changes and inflammatory cytokine analysis. Bronchoalveolar lavage fluid (BALF) was collected for total cell counts and protein concentration. Macrophages and neutrophils infiltration in the lungs was quantified via immunofluorescence staining. Four segments of the intestinal tract (jejunum, ileum, cecum, and colon) were harvested for histological analysis using hematoxylin and eosin (H&E), Alcian blue-periodic acid-Schiff (AB-PAS), and toluidine blue (TBO) staining. These evaluations included measurements of villus height to crypt depth, intestinal injury scoring, and counts of goblet and mast cells.

**Results:**

AKK-MC treatment resulted in higher fecal abundance of AKK compared to AKK group. AKK treatment attenuated LPS-induced weight loss and mitigated lung damage. This was evidenced by reduced protein concentration and cell counts in BALF, downregulation of *Tnf-α* and *Il-1β* expression, improved lung histology, and decreased macrophage infiltration and neutrophil extracellular traps formation. In the intestine, AKK treatment restored mucosal architecture, increased villus height to crypt depth ratios, maintained goblet cell populations, and reduced mast cell infiltration across intestinal segments.

**Conclusion:**

These results demonstrate that microencapsulation enhances AKK’s efficacy in ameliorating LPS-induced ALI in juvenile mice through gut microbiota modulation. This study provides a crucial foundation for the development of probiotic-based interventions in pediatric ALI.

## 1 Introduction

Acute lung injury (ALI) and its severe manifestation, acute respiratory distress syndrome (ARDS), are prevalent and critical respiratory syndromes. These conditions are characterized by dysregulated inflammation, injury, and coagulation processes both in the lung and systemically (Bos and Ware, 2022). While ALI/ARDS arises from heterogeneous etiologies, current clinical management primarily focuses on diagnosing and treating underlying causes, providing respiratory support, meticulous fluid management, and general supportive care (Matthay et al., 2019). Due to the complex pathophysiology and substantial mortality associated with ARDS, there is an urgent need for innovative therapeutic interventions.

Pediatric acute respiratory distress syndrome (PARDS), while sharing comparable pathophysiological mechanisms with adult ARDS, presents unique challenges due to variations in etiology, immune responses, and complications, contributing to its heterogeneity (Kneyber et al., 2023). Although PARDS occurs less frequently and with lower mortality than adult ARDS, its impact on pulmonary development and function in early childhood can lead to lasting respiratory sequelae, thus shaping unique long-term respiratory health trajectories for affected individuals (Bernard et al., 1994; Ranieri et al., 2012; Bellani et al., 2016). Therefore, the development of precision treatment strategies specifically for PARDS is of critical clinical importance.

The intestine serves as a vital immune organ and significantly influences physiological responses in distant organs, notably via the gut-lung axis (Ziaka and Exadaktylos, 2024). Gut microbiota are key regulators of this gut-lung cross-talk (Narayana et al., 2023). In ALI, gut microbial dysbiosis compromises intestinal barrier integrity, leading to increased permeability and immune dysfunction (Xie et al., 2024). This compromised barrier allows the translocation of harmful microbial products—such as lipopolysaccharide (LPS), bacterial metabolites, and pathogen-associated molecular patterns (PAMPs), into the systemic circulation. This translocation consequently exacerbates pulmonary inflammation (Wang et al., 2023).

The gut microbiome exhibits dynamic changes throughout life, influenced by diet, environment, and age, with the most pronounced developmental shifts occurring during childhood (Yatsunenko et al., 2012). While adults over 40 often display increased microbial diversity and a higher abundance of *Blautia* (Radjabzadeh et al., 2020), the infant gut microbiota is characterized by low diversity and enrichment in genes for lactose metabolism. The introduction of solid food is associated with a progressive increase in *Bacteroidetes*, a phylum adept at digesting plant polysaccharides (Koenig et al., 2011). Similarly, in juvenile mice, the early-life gut microbiota is relatively simple and progresses towards an adult-like composition following weaning and the initiation of solid food intake, marked by increasing microbial diversity and shifts in community structure (Kennedy et al., 2025).

*Akkermansia muciniphila* (AKK) is an anaerobic gut bacterium implicated in various health conditions, including obesity, non-alcoholic fatty liver disease, and inflammatory disorders (Depommier et al., 2019; Kim et al., 2020; Wang et al., 2020). AKK is particularly specialized in mucin degradation, playing a critical role in maintaining intestinal barrier integrity and regulating immune responses (Bakshani et al., 2025; Ioannou et al., 2025). Furthermore, studies have shown that AKK intervention attenuates LPS-induced ALI, preserves intestinal barrier function, and restores disrupted microbial communities (Shen et al., 2023). However, the specific role of AKK in juvenile mice remains to be fully elucidated.

Oral administration of exogenous microorganisms is highly vulnerable to environmental oxygen and harsh gastrointestinal conditions (Yao et al., 2020). Microencapsulation technology effectively shields probiotics from these adverse environments, thereby improving their storage stability, survival rate, and colonization efficiency. Despite the development of various microencapsulated probiotic formulations, research on AKK, particularly in the context of ALI, is scarce and largely unexplored.

In this study, the protective effects of AKK supplementation against ALI and preserve intestinal barrier integrity were investigated. Furthermore, microencapsulation was used to enhance AKK’s functional activity and viability. These findings illuminate the importance of gut-lung axis communication in ARDS and support the potential of probiotic-based therapeutics for PARDS, offering novel insights for precise treatment strategies. This study is the first to assess and compare the alleviative effects of microencapsulated AKK versus live AKK on ALI in a juvenile mouse model. Furthermore, AKK’s segment-specific regulatory impact on gut barrier function is elucidated, providing critical experimental evidence for targeted probiotic interventions in children.

## 2 Methods

### 2.1 Animal and modeling method

All animal experiments were conducted in strict accordance with guidelines approved by the Institutional Animal Care and Use Committee (IACUC) of the Chongqing Medical University (Approval No.: IACUC-CQMU-2023-0221). Male C57BL/6J mice, aged 3–4 weeks, were obtained from Chongqing Ensi Biotechnology Co., Ltd. (Chongqing, China). The mice were housed in a controlled environment with a 12 h/12 h light/dark cycle, ambient temperature maintained at 23 ± 1°C, and relative humidity at 50 ± 10%. Animals had ad libitum access to standard laboratory diet and water throughout the study. For bacterial supplementation, mice received daily oral gavage for 14 days with either *Akkermansia muciniphila* (1×10^9^ CFU/mouse) or a vehicle comprising 20% (v/v) glycerol dissolved in PBS. Acute lung injury was induced via intratracheal instillation of LPS (1 mg/kg) three days prior to sampling. The experimental groups were as follows: (A) Vehicle+PBS group: Mice received oral gavage of the vehicle and intratracheal instillation of PBS, n=10. (B) Vehicle+LPS group: Mice received oral gavage of the vehicle and intratracheal instillation of LPS, n=11. (C) AKK+LPS group: Mice received oral gavage of *Akkermansia muciniphila* bacterial solution and intratracheal instillation of LPS, n=10. (D) AKK-MC+LPS group: Mice received oral gavage of the microencapsulated *Akkermansia muciniphila* preparation and intratracheal instillation of LPS, n=11.

### 2.2 Bronchoalveolar lavage fluid collection and analysis

Bronchoalveolar lavage fluid (BALF) was collected and centrifuged 2,000 ×g for 10 mins at 4°C to separate the supernatant from the cell pellet. The cell pellet was treated with red blood cell lysis buffer (R1010, Solarbio) to lyse erythrocytes. After lysis, the remaining cells were resuspended in 1 mL of 0.9% sterile physiological saline. Total cell counts in BALF were determined using the Automated Cell Counter (C100-SE, RWD, Shenzhen, China). Protein concentration in the BALF supernatant was quantified with an Enhanced BCA Protein Assay Kit (P0010, Beyotime) according the manufacturer’s instructions.

### 2.3 *Akkermansia muciniphila* culture

The study strain of *Akkermansia muciniphila* (B336076, Mingzhoubio; original strain number: ATCC BAA-835) was cultured anaerobically at 37°C in Brain Heart Infusion Broth (BHI, MZM0090, Mingzhou Bio) supplemented with 0.5% porcine mucin (M2378, Sigma-Aldrich) and 0.05% L-cysteine (C7352, Sigma-Aldrich). Bacterial concentration was determined by measuring the optical density at 600 nm (OD_600_).

### 2.4 *Akkermansia muciniphila* sequencing

Genomic DNA was extracted from *Akkermansia muciniphila* following culture enrichment in BHI broth. Sequencing was performed by Sangon biotech (Shanghai, China). The resulting 50–550 bp sequences were used for Nucleotide Blast searches against the NCBI database (https://blast.ncbi.nlm.nih.gov/). Phylogenetic analysis was constructed based on aligned sequences using MEGA software (version 12).

### 2.5 *Akkermansia muciniphila* quantification

Fecal samples were stored at -80 °C. Genomic DNA was extracted using a Fecal Genome DNA Extraction Kit (DP328-02, Tiangen) according to the manufacturer’s protocol. DNA concentration was normalized to 100 ng/μl. Quantitative analysis was performed via real-time PCR using SYBR qPCR Master Mix (Q311-03, Vazyme) and the following primer sets: *Akkermansia muciniphila* (AKK-F: CAGCACGTGAAGGTGGGGAC; AKK-R: CCTTGCGGTTGGTCAGAT) and 16S rRNA (16srRNA-F: ACTCCTACGGGAGGCAGCAGT; 16srRNA-R: ATTACCGCTGCTGGC).

### 2.6 Encapsulation and morphological characterization of *Akkermansia muciniphila*

*Akkermansia muciniphila* was mixed in equal volumes with a 1.5% sodium alginate solution (S100126, Aladdin). The mixture was extruded as droplets into 0.1 M CaCl_2_ solution to form the primary alginate microcapsule layer. After 1-hour hardening period at room temperature, the capsules were washed with sterile physiological saline. A secondary chitosan layer was applied by immersion in 4% chitosan solution (C105799, Aladdin), followed by a third layer of 1% acacia solution (A108975, Aladdin). The microcapsules were collected by filtration, rinsed with PBS, and lyophilized for 12 hours. For scanning electron microscopy (SEM) analysis, freeze-dried microcapsules were mounted on conductive adhesive tape, gently air-dried to remove loose particles, and sputter-coated with gold. Morphological characterization was performed using a Field Emission SEM SU8010 (HITACHI, Tokyo, Japan).

### 2.7 Histology and immunofluorescence

After modeling, lung and intestine tissues were collected and fixed into 4% paraformaldehyde. Tissues were embedded in paraffin, sectioned, and stained. Images were captures by Olympus IX53 microscope (Olympus, Tokyo, Japan). After hematoxylin-eosin staining (H&E staining), lung injury scores were obtained through observing 5 aspects:(A) Neutrophils in alveolar space;(B) Neutrophils in interstitial space;(C) Hyaline membrane;(D) Proteinaceous debris filling in airspace; and (E)Alveoli septal thickening under 400x microscope. Lung injury scores =[(20×A) + (14×B) + (7×C) + (7×D) + (2×E)]/(number of fields ×100) (Matute-Bello et al., 2011). Colon and cecum section injury scores was conducted by optical microscopy and scored based on the extent of inflammatory infiltrate (0–5), crypt damage (0–4), ulceration (0–3), and edema (0–2) (Sun et al., 2024). Villus height and crypt depth were measured using ImageJ software (version 1.54f) based on images captured under a 20× objective lens (Zhao et al., 2024). The villus height/crypt depth was calculated as the villus height relative to the crypt depth. After Alcian Blue-Periodic Acid-Schiff (AB-PAS) staining, goblet cells were quantified by examining ten crypts per intestinal section (Yang et al., 2022). After Toluidine Blue O (TBO) staining, Mast cells were counted in 6 different randomly selected fields of view under 40× magnification and expressed as the number of cells per high power field (hpf) (Gao et al., 2026).

For immunofluorescence (IF), tissue sections were incubated with primary antibodies against CD68 and MPO (Proteintech, Wuhan, China). Imaging was performed using a Nikon A1R confocal microscope (Tokyo, Japan). CD68-positive cells were counted, and MPO-positive area was quantified under a 60× oil immersion objective.

### 2.8 Quantitative real-time PCR

Total RNA was extracted using the Eastep^®^ Super Total RNA Extraction Kit (LS1040, Promega). Subsequently, total RNA was reverse transcribed into cDNA using the Transcription Mix (A2801, Promega). Quantitative real-time PCR (qPCR) was performed on a CFX Connect™ Real-Time System (Bio-Rad, Hercules, CA, USA). Each 10-μL qPCR reaction mixture consisted 5 μL of SYBR qPCR Master Mix (Q311-03, Vazyme), 3.6 μL of DEPC-treated water, 0.2 μL of forward primer (10 μM), 0.2 μL of reverse primer (10 μM), and 1 μL of cDNA template. Relative mRNA expression levels were calculated using the 2^(-ΔΔCt) method, with *β-actin* serving as the reference gene for normalization. The primer sequences used were as follows. Mouse inflammatory factors: *Il-1*(Il-1-F, GGACAGAATATCAACCAACAA; Il-1-R, TTACACAGGACAGGTATAGATT), *Tnf-α* (Tnf-α-F, TCTCAGCCTCTTCTCATTC; Tnf-α-R, GCCATTTGGGAACTTCTC), the internal control gene *β-actin* (β-actin-F, GGCTGTATTCCCCTCCATCG; β-actin-R, CCAGTTGGTAACAATGCCATGT).

### 2.9 Statistical analysis

Data are presented as the mean ± standard error of the mean (SEM). For comparisons between multiple groups, one-way analysis of variance (ANOVA) was performed, followed by Tukey’s *post hoc* test. Changes in body weight over time were analyzed using two-way ANOVA with Dunnett’s multiple comparisons test. Nonparametric Kruskal–Wallis test was employed for data that did not meet normality assumptions. All *p*-values are reported directly within the figures. Statistical analyses were conducted using GraphPad Prism (version 10.1.2).

## 3 Results

### 3.1 Identification and microencapsulation of the AKK strain

**Figure 1:**
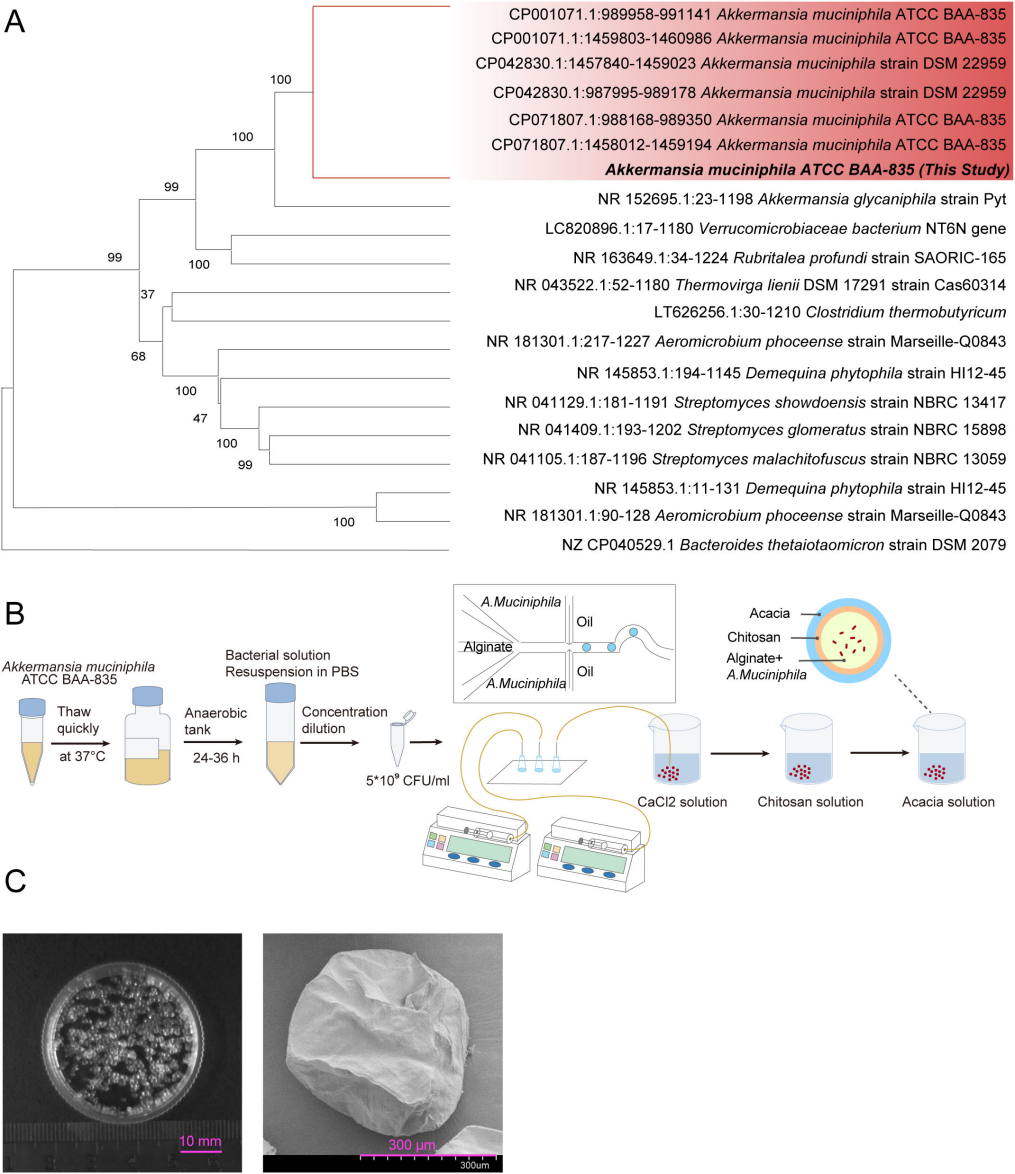
Fabrication and morphological characterization of AKK-MC. **(A)** Phylogenetic tree was constructed based on the sequencing results of AKK species. **(B)** AKK Culture Process and Microencapsulation Process. **(C)** Microcapsule appearance under OM and SEM. Scale bar, OM, 10mm; SEM, 300μm.

In this study, *Akkermansia muciniphila* ATCC BAA-835 (AKK) was employed. Whole-genome sequencing confirmed the absence of contaminating species (**Figure 1A**). Recognizing that oral administration of live bacteria can result in significant degradation by gastric and intestinal secretions, thereby reducing viability and colonization efficiency, microencapsulation was utilized to develop a protective AKK formulation (AKK-MC) (**Figure 1B**). Optical microscopy (OM) revealed uniform microcapsules with an approximate diameter of 1 mm, and scanning electron microscopy confirmed complete encapsulation of the bacterial cells within these microcapsules (**Figure 1C**).

### 3.2 Colonization of AKK and maintenance of body weight in juvenile mice

**Figure 2:**
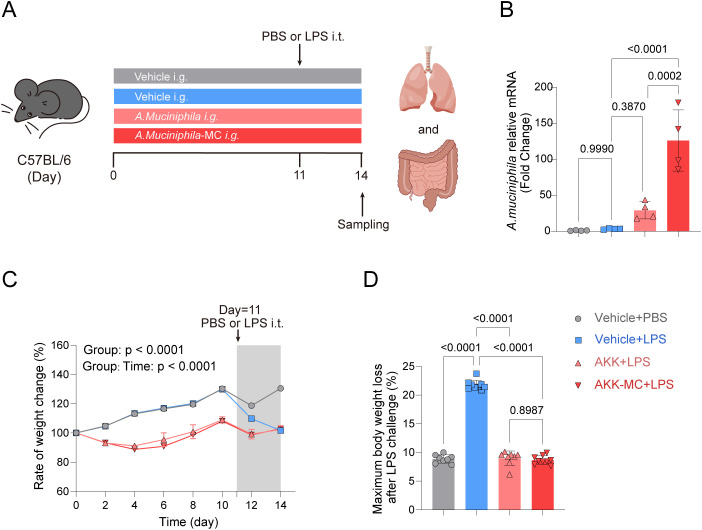
AKK-MC enhances colonization efficiency in juvenile mice intestine. **(A)** Experimental design and grouping. Mice were divided into four groups: Vehicle+PBS, Vehicle+LPS, AKK+LPS, AKK-MC+LPS. Mice were gavage with bacteria or vehicle for 14 days, intratracheal instillation of PBS or LPS on day 11, euthanasia on day 14 and collection of relevant materials. **(B)** Feces were collected on day 14 for qPCR relative quantification (*n* = 4). **(C)** The body weight of mice was measured from day 0 to day 14 (*n* = 10-11). **(D)** The ratio of maximum weight loss before and after LPS treatment (*n* = 10-11). One-way ANOVA with Tukey’s multiple comparisons test was used to calculate *p*-value in **(B)** and **(D)**. Two-way ANOVA with Dunnett’s multiple comparisons test was used to calculate p-value in **(C)**. Mean ± SEM.

Mice were orally administered live AKK or AKK-MC for two weeks prior to intratracheal instillation (i.t.) of lipopolysaccharide (LPS) to induce acute lung injury (ALI) (**Figure 2A**). In vehicle-treated controls, intestinal AKK abundance remained low. In contrast, oral gavage of AKK significantly enhanced intestinal colonization, with AKK-MC demonstrating even higher colonization efficiency (**Figure 2B**). Throughout the experimental period, analysis of the body weight curves indicated that both AKK colonization and LPS treatment influenced mouse body weight (**Figure 2C**). A two-way repeated measures ANOVA of body weight changes over time revealed a significant interaction between treatment and time (F (21, 210) = 236.4, p < 0.0001), signifying distinct body weight change trajectories across the four treatment groups. Furthermore, the maximum weight loss ratio, calculated from pre- and post-LPS administration measurements, demonstrated that AKK colonization mitigated LPS-induced weight loss (**Figure 2D**).

### 3.3 AKK and AKK-MC alleviate LPS-induced lung injury in juvenile mice

**Figure 3:**
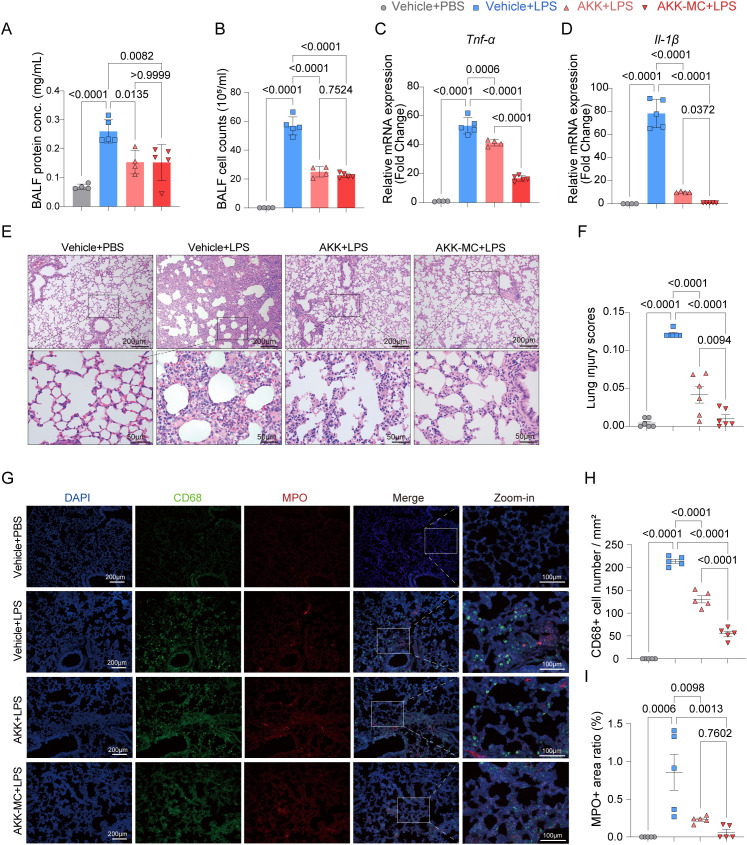
AKK and AKK-MC formulation alleviate phenotype of LPS-induced ALI. **(A)** Protein concentrations in BALF (*n* = 4-5). **(B)** Cell count in BALF (*n* = 4-5). **(C)***Tnf-α* mRNA relative expression in lung tissue (*n* = 4-5). **(D)***Il-1β* mRNA relative expression in lung tissue (*n* = 4-5). **(E)** H&E staining of lung tissue. Scale bar, 200μm, 50μm. **(F)** Lung injury score (*n* = 6). **(G)** Lung tissue immunofluorescence. Scale bar, 200μm and 100μm. **(H)** CD68 positive cells per square millimeter (*n* = 5). **(I)** MPO positive area ratio (*n* = 5). One-way ANOVA with Tukey’s multiple comparisons test was used to calculate *p*-value in **(A–D, F, H)** and **(I)**. Mean ± SEM.

Neutrophil infiltration following LPS challenge was assessed by measuring protein concentration and cell counts in bronchoalveolar lavage fluid (BALF). AKK colonization significantly attenuated cellular infiltration and protein leakage into the alveolar space, though no distinct formulation-dependent effect was observed (**Figures 3A, B**). Evaluation of inflammatory cytokine expression in lung tissue revealed that AKK colonization reduced the relative mRNA levels of *Tnf-α* and *Il-1β*. This inhibition is associated with the microencapsulation of AKK (**Figures 3C, D**). Histological examination via H&E staining and immunofluorescence further demonstrated that AKK mitigated lung tissue damage, reduced macrophage recruitment and inhibited neutrophil extracellular traps (NETs) formation (**Figures 3E–I**). AKK-MC exhibited enhanced efficacy in ameliorating these pathological phenotypes. Collectively, these results indicate that AKK colonization alleviates LPS-induced ALI, evidenced by improvements in histological architecture and immunomodulation. A correlation was observed between bacterial microencapsulation and the extent of phenotypic protection.

### 3.4 AKK and AKK-MC protect against LPS-induced intestinal injury in juvenile mice

**Figure 4:**
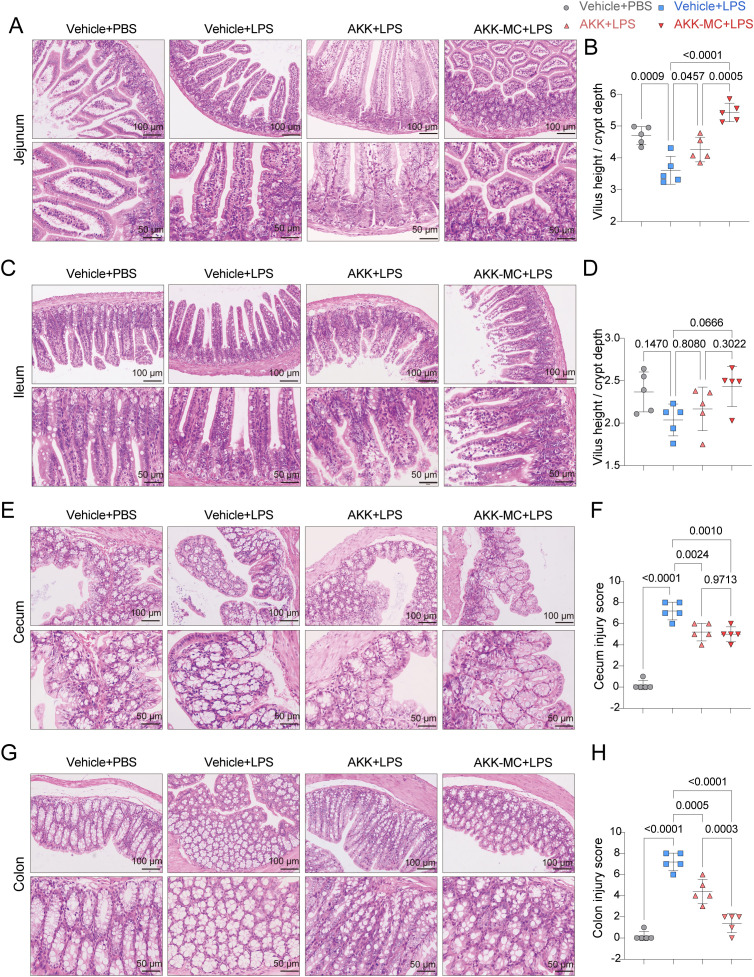
AKK and AKK-MC formulation alleviate LPS-induced intestinal histological damage. **(A)** H&E staining of jejunum tissue. Scale bar, 100μm, 50μm. **(B)** Jejunal villi length to crypt depth (V/C) (*n* = 5). **(C)** H&E staining of ileum tissue. Scale bar, 100μm, 50μm. **(D)** Ileal villi length to crypt depth (V/C) (*n* = 5). **(E)** H&E staining of cecum tissue. Scale bar, 100μm, 50μm. **(F)** Cecal injury score (*n* = 5). **(G)** H&E staining of Colon tissue. Scale bar, 100μm, 50μm. **(H)** Colon injury score (*n* = 5). One-way ANOVA with Tukey’s multiple comparisons test was used to calculate *p*-value in **(B)**, **(D)**, **(F)** and **(H)**. Mean ± SEM.

To assess the protective effects of AKK against ALI-induced intestinal injury, the morphological integrity of the jejunum, ileum, cecum, and colon was evaluated. The jejunum and ileum are characterized structurally by villi and crypts, which are sensitive indicators of intestinal injury. AKK colonization significantly restored villus architecture and reduced inflammatory cell infiltration in both small intestinal segments compared to the LPS group (**Figures 4A–D**). Furthermore, AKK administration normalized the villus height to crypt depth (V/C) ratio in the jejunum (**Figure 4B**). In the large intestine, colon and cecum, which lacks villi and exhibits deeper crypts, AKK colonization resulted in reduced goblet cell vacuolation (**Figures 4E, G**) and significantly lower histological injury scores compared to LPS-treated mice (**Figures 4F, H**), thereby mitigating indirect intestinal damage. Together, these findings demonstrate that AKK preserves intestinal barrier integrity by maintaining small intestinal villus and crypt morphology, reducing inflammatory cell infiltration, and alleviating damage in the large intestine.

### 3.5 AKK and AKK-MC protect intestinal mucus integrity in juvenile mice

**Figure 5:**
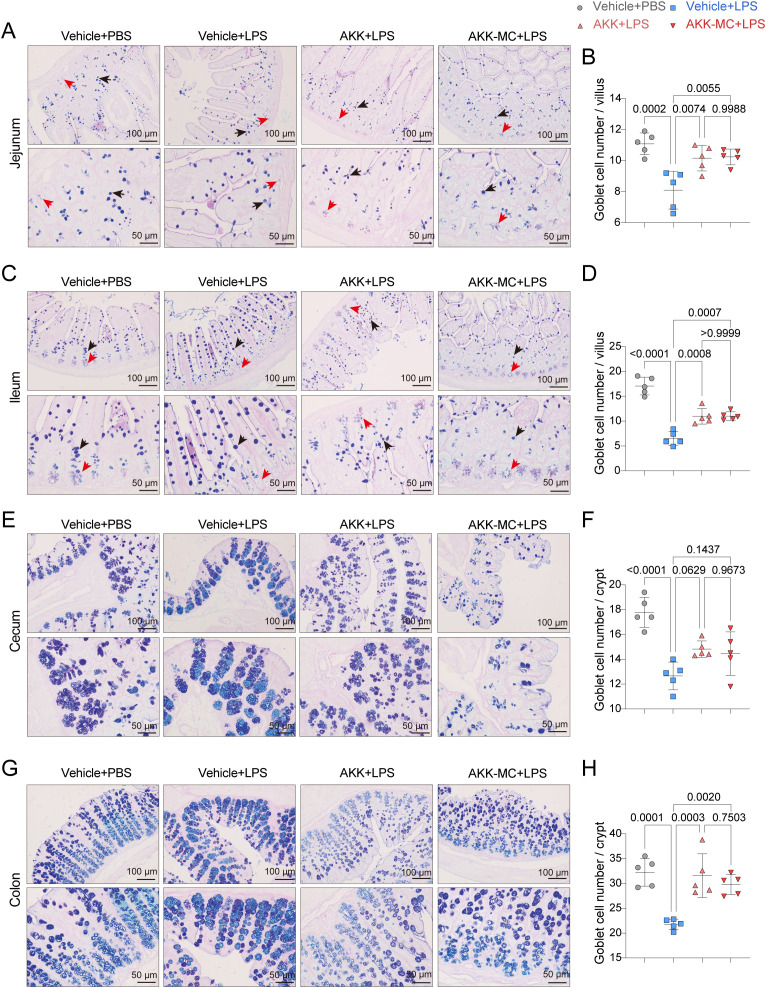
AKK and AKK-MC formulation restore LPS-induced intestinal goblet cells. **(A)** AB-PAS staining of jejunum tissue. Scale bar, 100μm, 50μm. **(B)** Goblet cell number per crypt in jejunum tissue (*n* = 5). **(C)** AB-PAS staining of ileum tissue. Scale bar, 100μm, 50μm. **(D)** Goblet cell number per crypt in ileum tissue (*n* = 5). **(E)** AB-PAS staining of cecum tissue. Scale bar, 100μm, 50μm. **(F)** Goblet cell number per crypt in cecum tissue (*n* = 5). **(G)** AB-PAS staining of colon tissue. Scale bar, 100μm, 50μm. **(H)** Goblet cell number per crypt in colon tissue (*n* = 5). One-way ANOVA with Tukey’s multiple comparisons test was used to calculate *p*-value in **(B)**, **(D)**, **(F)** and **(H)**. Mean ± SEM.

Goblet cells, specialized intestinal epithelium cells, primarily secrete mucus rich in acidic mucins. Paneth cells, situated at the base of small intestinal crypts, produce antimicrobial peptides and other proteins stored within alkaline mucus. The distributions of goblet cells and Paneth cells in the small intestine were assessed using AB-PAS staining. In the jejunum and the ileum, the LPS group exhibited significantly reduced goblet cell numbers and hypertrophic goblet cells. AKK colonization restored goblet cell numbers, normalized their morphology, and increased Paneth cell counts (**Figures 5A-D).** In the large intestine, AB-PAS staining revealed goblet cells containing acidic mucins (stained blue-purple) and mixtures of acidic and neutral mucins (blue). LPS challenge reduced goblet cell numbers in the cecum, resulting in large, densely packed mucin vesicles within crypts with acidic and mixed mucins arranged alternately. Both the AKK and AKK-MC groups showed reduced mucin vesicle density and increased goblet cell numbers (**Figures 5E, G**). However, AKK colonization restored goblet cell numbers and distribution, and this effect was independent of the AKK formulation used. (**Figures 5F, H**). Collectively, AKK regulates goblet cells and Paneth cell distribution in the small intestine and modulates mucin composition in the large intestine, thereby contributing to the stabilization of the intestinal microenvironment and mucosal integrity in juvenile mice with ALI.

### 3.6 AKK and AKK-MC reduce intestinal immune response in juvenile mice

**Figure 6:**
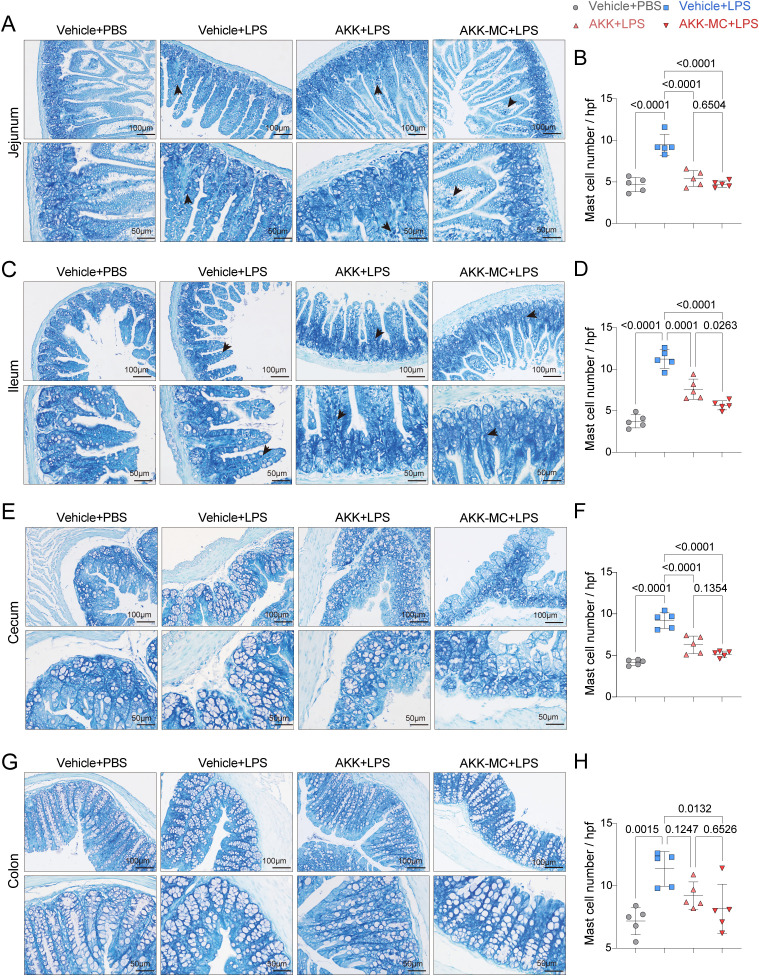
AKK and AKK-MC formulation reduce LPS-induced intestinal mast cell infiltration. **(A)** TBO staining of jejunum tissue, scale bar, 100μm, 50μm. **(B)** Mast cell number in jejunum tissue (*n* = 5). **(C)** TBO staining of ileum tissue, Scale bar, 100μm, 50μm. **(D)** Mast cell number in ileum tissue (*n* = 5). **(E)** TBO staining of cecum tissue, Scale bar, 100μm, 50μm. **(F)** Mast cell number in cecum tissue (*n* = 5). **(G)** TBO staining of colon tissue. Scale bar, 100μm, 50μm. **(H)** Mast cell number in colon tissue (*n* = 5). One-way ANOVA with Tukey’s multiple comparisons test was used to calculate *p*-value in **(B)**, **(D)**, **(F)** and **(H)**. Mean ± SEM.

Mast cells, critical immune effector cells in the intestine, are known to proliferate and activate during allergic and inflammatory responses, with the ileum typically exhibiting the highest mast cell density. Histological analysis of TBO-stained sections revealed prominent mast cell aggregation in the LPS group, often localized adjacent to goblet cells (**Figures 6A, C, E, G**). AKK colonization significantly reduced mast cell accumulation across all intestinal segments examined, an effect that was independent of AKK abundance (**Figures 6B, D, F, H**). These results indicate that AKK attenuates intestinal mast cell proliferation and activation. This mechanism may be instrumental in disrupting the detrimental gut-lung axis during ALI.

## 4 Discussion

**Figure 7:**
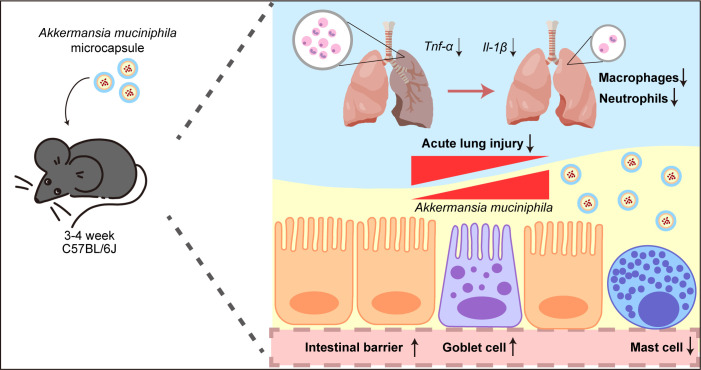
AKK and AKK-MC formulation alleviate LPS-induced ALI through the gut-lung axis in juvenile mice. AKK-MC enhances intestinal colonization and barrier function, promoting goblet cell activity and reducing mast cell infiltration. Amelioration of LPS-induced lung injury through attenuation of pulmonary inflammation.

In this study, juvenile mice were pretreated with live AKK or AKK-MC prior to ALI induction to evaluate AKK’s influence on lung and intestinal inflammatory (**Figure 7**). Microencapsulation effectively shield AKK from the gastrointestinal environment, leading to significantly higher fecal AKK abundance in the AKK-MC group, confirming enhanced colonization efficiency. AKK-MC treatment significantly improved intestinal colonization and effectively mitigated inflammatory processes in both organs. Specifically, AKK-MC reduced lung inflammatory mediator expression, preserved goblet cell morphology and function, and attenuated gut mast cell accumulation. These findings provide compelling evidence that AKK ameliorates ALI via the gut-lung axis, elucidate its protective intestinal mechanisms, and support the potential of probiotic-based interventions in juvenile ALI.

The establishment and maturation of the gut microbiome from early life play a critical role in shaping the developing immune system. Neonatal gut microbiomes are initially characterized by higher beta diversity and lower alpha diversity, gradually stabilizing and increasing in complexity to adult levels over time (Koenig et al., 2011; Bäckhed et al., 2015). In healthy adults, the *Firmicutes* and *Bacteroidetes* phyla form the dominant microbial communities (Ottman et al., 2012), contributing to stable microecological balance (Nicholson et al., 2012). Early life microbial succession involves a compositional shift, with newborns initially dominated by *Proteobacteria* and *Actinobacteria*, progressing to *Bifidobacterium* and *Collinsella* dominance by six months (Dogra et al., 2015). Crucially, the early life microbiota is indispensable for normal immune system development; its absence results in aberrant immune maturation (Gensollen et al., 2016). Functional contributions include the production of indole derivatives by *Bifidobacteria* from human milk oligosaccharides, which modulate CD4+ T cell differentiation and reduce inflammatory cytokine production (Henrick et al., 2021; Laursen et al., 2021). Furthermore, microbiota-derived SCFAs and antigens promote RORγt+ Treg cells that suppress intestinal inflammation and TH2 responses (Ohnmacht et al., 2015). The absence of a microbiota in germ-free mice leads to severe immunodeficiency, including reduced RORγt+ Treg cells, absent TH17 cells (Gensollen et al., 2016), and impaired antibody production (Donald and Finlay, 2023). In this study, juvenile mice were treated by AKK before LPS-induced ALI, not only verifying AKK’s protective efficacy through the gut-lung axis in early life but also providing crucial experimental support for microbiota intervention strategies in the prevention and treatment of PARDS.

Selective oropharyngeal decontamination (SOD) and selective digestive decontamination (SDD) are cornerstone strategies for preventing ventilator-associated pneumonia (VAP) and sepsis in intensive care units (ICUs) (Bos et al., 2017). Despite meta-analyses demonstrating reduced infection rates and mortality with SDD in mechanically ventilated ICU patients (Stoutenbeek et al., 1984; Hammond et al., 2022), their broad implementation is hampered by significant concerns. These include the potential induction of antibiotic resistance, the detrimental disruption of beneficial commensal flora, and uncertain long-term ecological impacts on the microbiome (Benus et al., 2010). The implications are particularly critical for pediatric populations, as critically ill children may display exaggerated responses to SDD (Kean et al., 2024), and early-life antibiotic exposure can profoundly alter gut microbiota composition and function (Bogaert and van Schaik, 2024). In this context, supplementation with specific probiotics, such as AKK, that possess well-defined protective roles offers a more favorable approach. These probiotics can actively and selectively bolster intestinal barrier integrity and immune homeostasis, thereby presenting a safer alternative to conventional decontamination methods.

The gut-lung axis represents a bidirectional regulatory network connecting the gastrointestinal and pulmonary systems through multifaceted mechanisms. Crucially, the integrity of the intestinal barrier is paramount for maintaining systemic immune homeostasis. Disruption of this barrier allows the translocation of bacterial products and endotoxins into the bloodstream, which can precipitate lung inflammation (Harkin et al., 2001; Lyons and Coopersmith, 2017) and subsequently contribute to ALI/ARDS. AKK, a probiotic, has demonstrated protective effects by enhancing intestinal tight junction proteins, reducing intestinal pro-inflammatory cytokines, and increasing anti-inflammatory cytokines (Jiang et al., 2025a). Furthermore, AKK has been shown to modulate signaling pathways such as IL-6/STAT3 and Wnt/β-catenin (Jiang et al., 2025b; Yang et al., 2026). Consequently, AKK supplementation is anticipated to ameliorate respiratory distress symptoms, such as ALI, through the regulation of gut-lung axis microecological balance. However, systematic investigations into the specific role and underlying mechanisms of AKK in pediatric populations remain scarce. This study therefore employs a juvenile mouse model to elucidate how AKK modulates the gut-lung axis to alleviate ALI, offering novel perspectives for the management of pediatric respiratory diseases.

Microencapsulated probiotics demonstrate enhanced survival and intestinal colonization rates within the gastrointestinal environment (Liu et al., 2026). Consistent with this, we detected a higher relative abundance of AKK in the feces of mice treated with AKK-MC versus the AKK bacterial solution. Weight change curves for these two groups largely overlapped and differed from the vehicle -only group early in gavage, suggesting AKK’s potential role in metabolic regulation (Xu et al., 2020; Zhang et al., 2021). After LPS treatment, all mice lost weight. Crucially, only the Vehicle group exhibited no weight gain. This indicates that the surgical procedure of tracheal instillation itself has an impact on mice, and the implantation of AKK can resist the damage caused by LPS.

ALI is characterized by the recruitment and activation of neutrophils within the pulmonary vasculature, leading to the release of pro-inflammatory cytokines that damage endothelial and epithelial barriers (Matthay and Zemans, 2011). This disruption of the alveolar-capillary barrier consequently permits protein leakage into alveolar spaces (Li et al., 2024). While this study did not observe a significant reduction in overall BALF cellularity or protein concentration with increased AKK abundance, AKK treatment markedly reduced macrophage infiltration and NETs formation. Both of these latter parameters showed a negative correlation with intestinal AKK levels. The observed “ceiling effect” for BALF cellularity and protein, contrasting with the dose-dependent improvement in inflammatory markers, strongly suggests that AKK protects against LPS-induced ALI primarily by safeguarding the intestinal barrier and reducing systemic endotoxin translocation, rather than through direct repair of the alveolar-capillary barrier. This gut-centric mechanism directly dampens the inflammatory cascade, explaining the clear dose-response in cytokines and immune cells. In contrast, the resolution of alveolar-capillary damage, as reflected by BALF protein and cells, appears to be an indirect consequence, plateauing once the primary systemic driver is sufficiently controlled. Collectively, these results indicate that oral administration of AKK confers substantial protection against LPS-induced lung injury, likely mediated through gut-based immunomodulatory mechanisms.

Since *Akkermansia* genus exhibits heterogeneous colonization, low in small intestine, high in large intestine (Luo et al., 2022), its effects on intestinal morphology and immunity were examined in four segments: the jejunum, ileum, cecum, and colon. Goblet cells are the primary producers of mucins, which are the key components of the mucus layer coating the intestinal epithelium (Gustafsson and Johansson, 2022). This mucus layer provides a physical barrier against luminal bacteria while serving as a niche for mucin-utilizing bacteria such as AKK (Paone and Cani, 2020; Wang et al., 2024). Notably, mucins serve as a carbon source for AKK, and in turn, AKK colonization can stimulate mucin production (Ioannou et al., 2025; Wang et al., 2025). Mast cells, acting as sentinel immune cells in the gut, respond to luminal or mucosal stimuli by releasing histamines and proteases (Gao et al., 2022). This release can contribute to visceral hypersensitivity and compromise barrier dysfunction (Bueno and Fioramonti, 2008; De Palma et al., 2022).

The intestinal barrier as a critical interface, separating the luminal environment from the underlying lamina propria and the mucosal immune system. This barrier is composed of a physical component, formed by intestinal epithelial cells (IECs), and a chemical component, comprising mucins and antimicrobial peptides secreted by specialized cells (Neurath et al., 2025). A compromised mucus layer allows for increased bacterial contact with the epithelium (Bergstrom et al., 2010), potentially causing epithelial damage or disruption of tight junctions (Horowitz et al., 2023). Such breakdown can permit bacterial translocation, a proposed mechanism for enteric flora invasion of the lungs during sepsis-induced ARDS (Dickson et al., 2016). IECs arise from stem cells at the crypt base, differentiating into specialized cell types, such as enterocytes, goblet cells, and Paneth cells. These cells migrate towards the villus tips, where aged cells are shed (Clevers, 2013). AKK has demonstrated its capacity to promote the proliferation and development of intestinal cells and organoids (Kim et al., 2021; Duan et al., 2023; Kang et al., 2024). It stimulates stem cell-mediated epithelial renewal and enhances the differentiation of Paneth and goblet cells, potentially through the production of SCFAs or activation of the *Wnt* signaling pathway by bacterial proteins. Accelerating mucin turnover during infection can aid in pathogen clearance and reinforce barrier function, thus representing a potential mechanism by which AKK alleviates intestinal inflammation (McLoughlin et al., 2016).

In addition to strengthening intestinal barrier integrity, AKK mitigates inflammation by modulating the host immune response. Toll-like receptors (TLRs), key sensors of microbial molecules, are central to this interaction (Janeway and Medzhitov, 2002). Specifically, AKK’s outer membrane protein Amuc_1100 has been shown to interact with Toll-like receptor 2 (TLR2), thereby contributing to the prevention of obesity and its sequelae (Plovier et al., 2017). Furthermore, a novel tripeptide Arg-Lys-His peptide, derived from AKK, demonstrates a protective effect against sepsis by binding to Toll-like receptor 4 (TLR4) (Xie et al., 2023). AKK’s secreted threonyl-tRNA synthetase engages with TLR2, promoting M2 macrophage polarization and the subsequent secretion of the anti-inflammatory cytokine IL-10 (Kim et al., 2023). In the adaptive immune compartment, AKK enhances immune responses mediated by RORγt+ regulatory T (Treg) cells, which are instrumental in suppressing autoimmunity and promoting tissue repair (Dikiy and Rudensky, 2023). This contributes to maintaining the critical proportion of Treg/Th17 cells (Ma et al., 2025). Moreover, AKK confers a training effect on innate immunity, leading to attenuated release of pro-inflammatory cytokines (Peña-Cearra et al., 2024).

While this study demonstrated the beneficial role of AKK in mitigating ALI via the gut-lung axis, several limitations warrant acknowledged. Firstly, as an endpoint study, the dynamic temporal progression between AKK colonization, gut barrier restoration, and subsequent phenotypic improvement could not be fully elucidated. Secondly, the observed protective efficacy of AKK was solely validated in an LPS-induced ALI model, raising questions about its generalizability to other ALI/ARDS etiologies and models. Thirdly, the study exclusively utilized male juvenile mice, limiting the generalizability of findings to female population. Finally, although we characterized AKK’s protective effects on intestinal barrier integrity, the precise molecular mechanisms underlying these effects remain incompletely understood. Identifying the active immunomodulatory metabolites produced by AKK and elucidating their specific molecular targets are critical next steps for future investigation. Subsequent research will incorporate genetic knockout models, comprehensive metabolomic profiling, and diverse ALI/ARDS animal models to thoroughly decipher the mechanistic underpinnings of AKK’s protective actions and to advance its clinical translation.

In summary, this study elucidates the critical role of AKK in maintaining goblet cell homeostasis, mitigating mast cell infiltration, and preserving intestinal barrier integrity in juvenile mice. Collectively, these mechanisms likely contribute to the alleviation of LPS-induced ALI via the gut-lung axis. Significantly, these findings were established in a juvenile model, a developmental stage characterized by heightened immune and microbial plasticity, thereby underscoring the potential for gut-lung axis to be uniquely responsive to probiotic intervention during early life. Furthermore, the microencapsulation technology developed herein effectively enhances AKK survival under challenging gastrointestinal conditions, presenting a robust strategy for improving probiotic delivery and laying the groundwork for clinical translation. These findings offer an experimental rationale for the potential application of AKK in mitigating pediatric ALI, support the development of pediatric-friendly microencapsulated probiotic formulations, and facilitate the integration of next-generation probiotics in childhood therapeutics.

## Data Availability

The original contributions presented in the study are included in the article/supplementary material. Further inquiries can be directed to the corresponding authors.
